# The Spatial Updating Mechanism of Different Field Cognitive Styles in Various Scene Layouts: Evidence from Behavior and fNIRS

**DOI:** 10.3390/bs16071125

**Published:** 2026-07-06

**Authors:** Ying Li, Xia Sun, Yu Liu, Yixue Dong

**Affiliations:** 1 Faculty of Psychology, Tianjin Normal University, Tianjin 300387, China; 2330340081@stu.tjnu.edu.cn (X.S.); 2530340010@stu.tjnu.edu.cn (Y.D.); 2 School of Psychology, Shaanxi Normal University, Xi’an 710119, China; lyu_victory@snnu.edu.cn

**Keywords:** field cognitive style, scene layout, spatial updating, reference frame, functional near-infrared spectroscopy (fNIRS)

## Abstract

Spatial updating—the ability to continuously revise spatial representations during locomotion—is fundamental to adaptive navigation and depends on flexible reference frames. Although previous research has established independent effects of field cognitive style and scene layout on spatial performance, their interaction and underlying neural substrates remain poorly understood. The present study examined how field dependence–independence and environment geometry jointly modulate spatial updating by combing the judgment of relative direction (JRD) paradigm with functional near-infrared spectroscopy (fNIRS). Forty participants were recruited and assigned to two groups (20 field-independent [FI] and 20 field-dependent [FD]) based on Embedded Figures Test scores. They completed directional pointing tasks in two virtual environments: a geometrically structured rectangular spaces affording explicit orthogonal reference axes and an ambiguous oval environments devoid of stable global geometric anchors. Behaviorally, FI individuals exhibited shorter response time in rectangular layouts yet superior accuracy in oval layouts relative to FD individuals. Neurally, the middle frontal gyrus (MFG) emerged as a critical locus exhibiting a significant interaction effect between cognitive style and environmental layout. Significant main effects of field cognitive style were observed in the precentral gyrus, superior parietal lobule, and paracentral lobule, with FI individuals showing greater oxyhemoglobin (HbO) elevation than FD participants. Collectively, these findings may tentatively suggest an interpretation that FI individuals flexibly alternate between internal egocentric and external allocentric reference frames during spatial information processing, whereas FD individuals predominantly rely on inherent structural cues embedded in the external environment. These findings may reflect cortical hemodynamic correlates of field cognitive style differences during spatial processing, and may offer empirical references for relevant cognitive neuroscience research and subsequent exploratory applications.

## 1. Introduction

During locomotion and navigation, humans must constantly update the perceived positions and directions of surrounding objects from their shifting personal perspective, a core cognitive capacity defined as spatial updating (Banta et al., 2011). With this fundamental capacity, observers actively reconstruct environmental spatial relations and dynamically bind positional associations between their own body and surrounding space, and such ongoing spatial computation is fundamentally hinged on the reference frame adopted by the navigating individual. Serving as the core anchoring framework for encoding self–environment spatial relationships, reference frames decisively shape the execution of spatial updating (Song et al., 2024). Recent theoretical frameworks indicate that spatial updating is not a unified automatic process but rather relies on the flexible integration of multiple reference systems (Zhao & Warren, 2024). Two predominant spatial coding modes have been well-established based on individual habitual strategies for spatial representation: the egocentric frame centers on the observer’s own body, while the allocentric frame depends on fixed external environmental landmarks and structural boundaries (R. F. Wang, 2017; Santoro et al., 2017). The most prominent divergence between them lies in their responses to bodily locomotion: the core axial orientation of egocentric representation shifts synchronously with physical displacement, whereas allocentric coordinates remain fixed independently of observer displacement. Accordingly, humans have to iteratively revise their self-location judgment based on perceived self-motion information, leading to substantial individual differences in updating strategies and spatial performance across varying environmental cue conditions.

Learning an environment’s layout and its spatial relations requires active navigation. The presence of clear reference directions influences the spatial frame a person selects. Salient external cues significantly facilitate the precision of spatial recognition (Lei et al., 2023), and bounded environments equipped with distinct landmarks enhance optic flow signals (Borodaeva et al., 2023). Spatial layouts can be encoded relative to an internal or an external frame. Individuals can also update their orientation from a fixed reference direction. Even when the test scene lacks a clear reference, a cognitive mechanism can recover the correct direction. This mechanism is sensitive to the spatial organization of the local environment, with room morphology representing a typical instantiation of such geometric constraints (L. Wang & Mou, 2020; Julian et al., 2018). Environmental geometric shape profoundly modulates spatial orientation. Rectangular scenes have distinctive orthogonal long and short axes, whose geometric characteristics facilitate the organization of inter-object spatial relationships (He et al., 2018). In contrast, a circular room without visible corners provides no such intrinsic reference information (Kelly et al., 2008).

The present study adopts rectangular and oval layouts to experimentally manipulate the availability of a stable allocentric reference frame. A rectangular boundary provides salient orthogonal axes (a clear long axis and a short axis) and right-angled corners, whose geometric features constitute a dependable external coordinate system to facilitate spatial alignment and directional orientation (He et al., 2018). In contrast, an oval layout—a smooth curved perimeter without straight wall segments or angular corners—lacks global geometric reference axes. Although identical landmark objects are placed in both layouts, the oval environment does not offer a well-defined environmental axis to anchor directional judgments. This forces individuals to rely more heavily on egocentric updating or landmark-based strategies (Kelly et al., 2008). By contrasting performance and neural activation between these two layouts, we can isolate how the presence versus absence of a clear allocentric frame interacts with individual differences in field cognitive style. This reliance on environmental structure produces the “alignment effect” (also called “orientation dependence”). Spatial performance improves when an imagined viewpoint aligns with a real or well-established environmental axis. However, the strength of this effect is not constant. It varies with how spatial knowledge is acquired and with individual characteristics.

Cognitive style constitutes a pivotal source of individual variation, profoundly shaping how people observe their surroundings and retrieve spatial information (Ishikawa, 2023; Giancola et al., 2023). A recent meta-analysis has confirmed its substantial effect on spatial navigation performance (Boccia et al., 2017). Among various cognitive style typologies, field dependence–independence (FDI), first proposed by Witkin et al. (1964), is the most extensively studied in spatial cognition. FDI directly reflects individual differences in perceiving and processing environmental and spatial information. Witkin (1977) defined FDI as the degree to which a person relies on external cues versus internal frames of reference. Field-independent (FI) individuals prefer internal bodily references for spatial computation, whereas field-dependent (FD) observers disproportionately depend on surrounding perceptual fields (Nori et al., 2023). This fundamental difference appears clearly during dynamic adjustments of spatial reference frames. In environments with fewer cues, FI individuals tend to perform more accurately. FD individuals may respond more quickly when cues are abundant (Tascón et al., 2017). Recent work on spatial–temporal contexts further demonstrates that spatial representations are not static but are dynamically shaped by both environmental features and internal task demands (Y. Wang & Zhang, 2025), reinforcing the notion that individual differences in cognitive style may modulate how spatial information is flexibly retrieved in updating tasks. Despite these insights, the precise mechanisms through which FDI modulates human navigation remain debated.

To explore these mechanisms, the judgment of relative direction (JRD) paradigm has been widely adopted and refined (Rieser, 1989). In this paradigm, participants learn an environment layout. They then point to target objects from an imagined position and orientation. A robust behavioral finding is the sensorimotor alignment effect. JRD performance is better when the imagined perspective aligns with the participant’s actual facing direction (R. F. Wang, 2017; Hatzipanayioti et al., 2016). Recent evidence indicates that this alignment effect is modulated by cognitive style (Nori et al., 2023). Yet interactive influences between FDI and environmental geometric features, alongside their underlying neural substrates, remain empirically unexamined.

Against this backdrop, the present study aims to bridge the gap between classic behavioral findings and contemporary neuroscience evidence by combining the JRD paradigm with functional near-infrared spectroscopy (fNIRS). fNIRS tolerates mild head movements efficiently, rendering it uniquely suitable for measuring cortical hemodynamic fluctuations during ecologically valid spatial cognitive tasks. Relative to functional magnetic resonance imaging (fMRI), fNIRS is constrained by moderate spatial resolution, limited penetration depth, and temporal sampling bounded by canonical hemodynamic delay; nevertheless, it provides adequate temporal sampling for detecting task-evoked cortical hemodynamic changes (Hagen et al., 2014; Yeo et al., 2023). Indeed, the applicability of fNIRS in spatial cognition research has been recently validated by Ou et al. (2025), who employed whole-head fNIRS, synchronized with eye-tracking, to reveal the neural correlates of spatial representation in orienteering athletes, demonstrating that fNIRS can effectively capture cortical activation patterns during dynamic spatial orientation tasks. Furthermore, fNIRS is a feasible and reliable neuroimaging technique for comparing cortical hemodynamic activation patterns among individuals with different cognitive styles (Yeo et al., 2023), owing to its good tolerance to slight head movement and high sensitivity to prefrontal–parietal cortical responses (Matsumoto et al., 2020). Regarding spatial coding, egocentric encoding produces significant activation in the right posterior inferior parietal lobe (Derbie et al., 2021). Allocentric encoding engages the left precentral gyrus and intraparietal sulcus. Both types of encoding elicit task-related hemodynamic activities in the right superior frontal gyrus and the postcentral gyrus (Heinzel et al., 2013). Accordingly, the present investigation restricts primary neural analysis to frontal and parietal lobes, core anatomical substrates subserving cognitive control and distributed spatial processing networks (Kang, 2023; Li et al., 2023).

Despite accumulating behavioral documentation delineating spatial updating mechanisms, a critical research void persists regarding synergistic interactions. The synergistic interplay between field cognitive style and environmental factors is still poorly understood. Its neural substrates are equally unclear. The individual effects of perspective orientation (He et al., 2018; Liu et al., 2016) and field cognitive style (Nori et al., 2023) on spatial performance are established. Yet their combined influence on the sensorimotor alignment effect and on patterns of cortical activation remains unexplored (Girolamo et al., 2024). Targeting three outstanding research questions, the present study addresses: (a) how field dependence–independence modulates spatial updating performance, (b) whether cognitive style interacts with the availability of environmental reference frames (i.e., scene layout), and (c) which fronto-parietal networks support this interplay. By examining this synergistic mechanism, we clarify how internal and external reference frames are weighted during self-motion. This work refines theoretical accounts of individual differences in environmental cue dependency. In addition, identifying the neural correlates of these interactions may provide an empirical basis for personalized cognitive training and inform the design of adaptive, user-centered navigation systems.

## 2. Materials and Methods

### 2.1. Participants

Participants were categorized into field-dependent (FD) and field-independent (FI) groups strictly based on scores obtained from the Embedded Figures Test (EFT), a standardized assessment developed by Witkin (1977) to quantify field cognitive style. The present study adopted the Chinese localized revision compiled by Zhang and Wang (1989) with an internal consistency reliability of 0.90. The test comprises 9 practice trials and 20 formal items, requiring participants to isolate predefined simple geometric figures embedded within complex graphic configurations under prescribed time constraints. All of the 105 college students (48 males, 57 females) completed this online test via the Questionnaire Star platform, and their EFT T-scores were used to classify individuals into field-independent (FI; T > 50) and field-dependent (FD; T < 50) groups for subsequent subject screening. Participants ranged in age from 17 to 24 years (*M* = 19.08, *SD* = 1.86), with EFT scores ranging from 34.58 to 72.79 (*M* = 52.58, *SD* = 8.74). For males, scores ranged from 34.58 to 72.79 (*M* = 51.81, *SD* = 9.71), for females, scores ranged from 36.32 to 66.99 (*M* = 53.22, *SD* = 7.86). This yielded 39 FD participants (20 males, 19 females) and 66 FI participants (28 males, 38 females). An extreme-group sampling strategy was implemented to maximize intergroup divergence in field cognitive style, thereby amplifying the behavioral and neural effects of cognitive style on spatial updating while eliminating confounding from intermediate EFT scores.

A power analysis was conducted using G*Power 3.1 (Faul et al., 2007). We adopted a conventional medium effect size of *f* = 0.25, which is widely employed in behavioral and spatial cognition studies involving field dependence–independence individual differences (Boccia et al., 2017; Nori et al., 2023). We set the α level at 0.05 and the statistical power at 0.80. The analysis indicated a minimum required sample size of 24 participants. We ultimately selected 40 participants—20 FI and 20 FD—all of whom completed the full experiment. Within the FI group, we selected the 10 highest-scoring males and the 10 highest-scoring females (total: *M* = 63.61, *SD* = 3.44; males: *M* = 63.80, *SD* = 4.39; females: *M* = 63.42, *SD* = 2.33). Within the FD group, we similarly selected the 10 lowest-scoring (total: *M* = 40.87, *SD* = 3.42; males: *M* = 39.45, *SD* = 3.00; females: *M* = 42.28, *SD* = 3.32). To further verify the validity of the selection, an independent-sample *t*-test was conducted on the EFT scores of the final 40 participants, revealing a significant between-group difference (*t*(38) = 10.258, *p* < 0.001, Cohen’s *d* = 6.63). Notably, the extremely large Cohen’s *d* value is attributed to our extreme-group sampling strategy, which deliberately selected participants with the highest and lowest EFT scores to maximize group differences in field cognitive style. This confirmed that the selected FI and FD participants represented two distinct cognitive style groups with robust differences. Among the final 40 participants, each group included 10 males and 10 females, achieving strict gender balance between FI and FD groups. An independent-sample *t*-test confirmed no significant age difference between FI and FD groups (*p* > 0.05). In subsequent behavioral and fNIRS statistical analyses, sex was included as a controlled covariate in exploratory analyses to rule out potential gender confounding effects on spatial updating performance and cortical activation. This sampling strategy is consistent with previous studies on field cognitive style (Boccia et al., 2017; Nori et al., 2023), which also adopted extreme score selection to identify prototypical FI/FD individuals and enhance the detectability of cognitive style effects. Notably, this extreme-group sampling approach effectively amplifies group-level behavioral and neural differences; however, it inevitably limits the generalizability of the present findings to individuals across the full continuum of field cognitive style, especially those with intermediate FDI scores. All enrolled participants were right-handed and executed behavioral responses using their dominant right hand to rule out performance bias originating from age-dependent functional variation in non-dominant limbs (Teixeira, 2008).

The study was conducted in accordance with the Declaration of Helsinki. The research protocol was reviewed and approved by the Tianjin Normal University Research Ethics Committee (Approval Number: 2025102306). All participants were informed about the study’s purpose and procedures and provided written informed consent prior to their participation.

### 2.2. Experimental Design

A 2 (Field Cognitive Style: FI, FD) × 2 (Scene Layout: Rectangular, Oval) mixed-factorial experimental design was employed. Field cognitive style was a between-subject variable, scene layout was a within-subject variable. To eliminate potential sequence effects, the order of the two layouts was counterbalanced across participants: half of the participants (10 FI, 10 FD) completed the rectangular layout first, followed by the oval layout, while the other half (10 FI, 10 FD) completed the oval layout first, followed by the rectangular layout. The study utilized both behavioral performance measures and functional near-infrared spectroscopy (fNIRS), with dependent variables including reaction time, accuracy rate, and mean HbO concentration.

#### 2.2.1. Experimental Scene Layout

**Figure 1:**
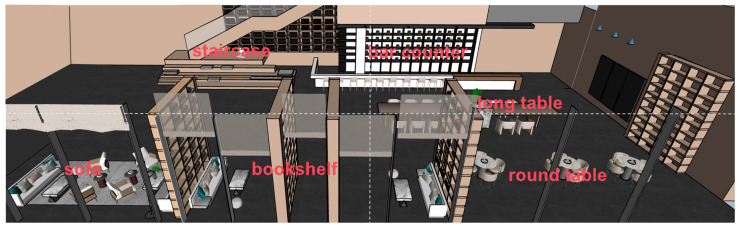
Rectangular scene layout with straight walls and right-angle corners, providing salient global orthogonal reference axes, with consistent landmark arrangement as the oval scene. Around the central starting platform, several landmark objects are arranged: a sofa, a bookshelf, a round table, a long table, a bar counter, and a staircase. Uneven wall coloring and wall-mounted decorative elements are merely cosmetic design elements of a 3D virtual scene without functional spatial cues. The six critical landmark objects were fully matched between rectangular and oval environments, and post-experiment subjective rating data confirmed participants relied solely on global geometric shape instead of local wall details during spatial judgment, so such visual differences cannot bias behavioral and neural results. (© 2026 by the authors).

**Figure 2:**
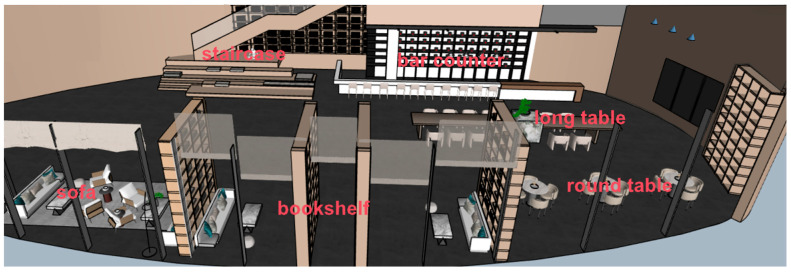
Oval scene layout with continuous curved boundaries and no angular corners, lacking global geometric reference axes, while retaining identical local landmark settings as the rectangular scene. Around the central starting platform, several landmark objects are arranged: a sofa, a bookshelf, a round table, a long table, a bar counter, and a staircase. Uneven wall coloring and wall-mounted decorative elements are merely cosmetic design elements of a 3D virtual scene without functional spatial cues. The six critical landmark objects were fully matched between rectangular and oval environments, and post-experiment subjective rating data confirmed participants relied solely on global geometric shape instead of local wall details during spatial judgment, so such visual differences cannot bias behavioral and neural results. (© 2026 by the authors).

Two virtual environments were constructed in Unity 3D (Unity Technologies, San Francisco, CA, USA) to instantiate the two levels of the scene-layout variable: a rectangular hall and an oval hall. Both halls contained a central open area with a slightly elevated starting platform, around which six distinctive landmark objects (a sofa, a bookshelf, a round table, a long table, a bar counter, and a staircase) were placed equidistantly. The critical manipulation concerned the shape of the outer boundary. The rectangular hall was bounded by straight walls with clear 90° corners, forming prominent orthogonal geometric axes and stable allocentric reference cues. By contrast, the oval hall adopted a smooth continuous curved boundary without straight wall segments or angular corners. Although both scenes contained identical landmark objects, the oval layout lacked global orthogonal geometric reference axes; local furniture landmarks existed but could not form a stable global directional reference frame. To empirically validate the intended manipulation of environmental reference frame availability, a post-experimental manipulation check was administered to all 40 participants immediately after the fNIRS session. Participants rated two statements on a 7-point Likert scale (1 = strongly disagree, 7 = strongly agree): (1) The rectangular hall provided clear directional cues (e.g., straight walls, corners) that helped me orient. (2) The oval hall provided clear directional cues. A paired-samples *t*-test revealed that participants rated the rectangular layout (*M* = 6.45, *SD* = 0.68) as providing significantly clearer directional cues than the oval layout (*M* = 2.35, *SD* = 1.02), *t*(39) = 20.67, *p* < 0.001, Cohen’s *d* = 4.78. This confirms that the oval layout was perceived as geometrically ambiguous with a significantly weaker environmental reference frame compared to the rectangular layout. Consequently, the oval layout lacks a well-defined environmental reference frame, making spatial orientation more ambiguous and likely promoting reliance on egocentric or landmark-based strategies. This systematic variation in boundary shape was designed to manipulate the availability of a reliable spatial reference frame: the rectangular scene provides a salient allocentric frame that aids direction judgment, whereas the oval scene provides a geometrically weak frame that does not readily support orientation. The experimental scene was presented as a 90 s video, created by a 360° rotation around the center point of the hall, and is depicted in Figure 1 (rectangular scene layout) and Figure 2 (oval scene layout).

#### 2.2.2. Experimental Task

**Figure 3:**
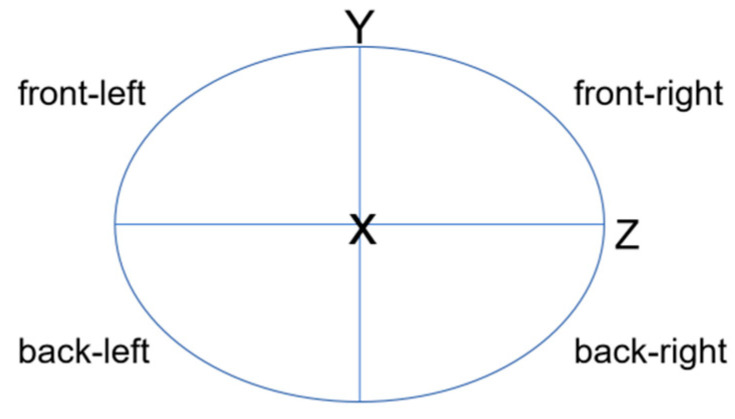
Example of the spatial updating experiment using the direction circle method (© 2026 by the authors).

The pointing task used the directional circle method (Figure 3), a well-established paradigm for assessing spatial updating and measuring knowledge of relative directions. In this method, participants viewed a circle diagram. They imagined standing at location X, marked at the center, and facing location Y, marked at the top. They then drew an arrow from the center of the circle (X) toward the target object. This arrow indicated the relative direction to the target object. The first two objects established the reference position and orientation, while the third was the target. The question was presented as follows: “Imagine you are at one location, facing another location. Please point toward the third location. In which direction is the third location relative to you?” Response options were: front-left, back-left, front-right, and back-right.

The pointing task in this study used four categorical response options (front-left, front-right, back-left, back-right) rather than continuous angular pointing. This design was intentionally chosen for three main reasons. First, to ensure compatibility with fNIRS measurement, discrete button responses (completion range: 500–800 ms) minimize confounding motor artifacts during concurrent fNIRS cortical monitoring. Second, to maintain comparability with classic judgment of relative direction (JRD) studies (Rieser, 1989; R. F. Wang, 2017; Nori et al., 2023), which have reliably demonstrated sensorimotor alignment effects and cognitive style differences using similar categorical direction judgments. Third, restricting response options reduces sustained cognitive load. Given that each participant completed 96 formal trials, this design effectively lowers response errors triggered by mental fatigue.

### 2.3. Procedure

Prior to participant setup, the NIRx recording system was interfaced with testing hardware, data acquisition equipment, and an EASYCAP optode holder. The optode layout, generated in NIRsite, was then imported into the Aurora 1.3 software. The experimenter assisted the participant in wearing the fNIRS device, and the formal experiment commenced once signal quality for most optodes reached an acceptable level. After the practice session, the screen went blank, and resting-state data were recorded for 1 min before proceeding to the formal experiment.

#### 2.3.1. Learning Phase

Participants viewed a 90 s video depicting a hall comprising six distinct areas: a sofa, a bookshelf, a round table, a long table, a bar counter, and a staircase. The video was created through a 360° rotation filmed from the central viewpoint of the hall. During viewing, participants encoded the spatial relationships between the central point of the scene and each of the six areas. The scene layout was randomly set as either rectangular or oval. To control potential confounding effects of learning exposure, we strictly recorded the number of video replays for each participant. An independent-sample *t*-test revealed that the average number of video viewing repetitions did not differ significantly between the FI group (*M* = 1.26, *SD* = 0.32) and the FD group (*M* = 1.22, *SD* = 0.29), *t*(38) = 0.416, *p* = 0.680. Participants were required to attain a minimum spatial memory accuracy threshold of 75% during practice trials before advancing to formal testing, which further standardized learning familiarity and minimized systematic differences in prior environmental encoding across groups. After confirming their memory, they were required to describe the spatial relationships between the central point and the six areas to the experimenter. Only participants achieving an accuracy rate exceeding 75% in the practice session proceeded to the formal experiment.

#### 2.3.2. Formal Experiment Phase

Participants watched a 90 s video depicting a hall composed of the same six areas. The video was created through a 360° rotation filmed from the central viewpoint of the hall. The video used in the formal experiment could differ from that in the practice session, primarily in its scene layout, requiring participants to carefully observe and memorize the spatial relationships between the central point and the six areas. Participants could watch the video multiple times until they correctly memorized the scene. Subsequently, the pointing task began.

**Figure 4:**
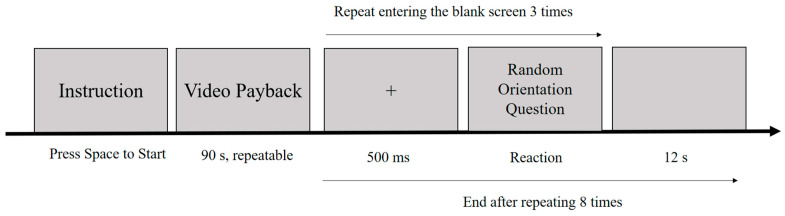
The experimental flowchart includes two phases: (1) a 90 s video playback for learning the scene layout, and (2) a random orientation question about the target object relative to the imagined position and facing direction (© 2026 by the authors).

The formal experiment consisted of 96 randomized trials in total. The task included 48 unique object–reference–target spatial combinations (e.g., “Imagine you are at the sofa, facing the bookshelf. Point to the round table”), which were evenly assigned across the two scene layouts, yielding 48 trials for the rectangular layout and 48 trials for the oval layout. Each unique combination appeared exactly once per scene layout. All trial sequences were fully randomized across participants and conditions using a computer-generated random sequence (Unity 3D random number generator). The four response options (front-left, back-left, front-right, back-right) were counterbalanced and presented with equal probability throughout the task to avoid response bias. Reaction time (RT) was quantified as the temporal interval from stimulus onset of the directional circle graphic to participants’ button-press responses, encapsulating all core cognitive processes including instruction comprehension. This RT duration covered the entire cognitive and behavioral process, including reading task instructions, mentally imagining the designated viewpoint, spatial updating reasoning, and making a directional choice. The entire experiment lasted approximately 25 min. The experimental procedure is illustrated in Figure 4.

### 2.4. fNIRS Data Acquisition

**Figure 5:**
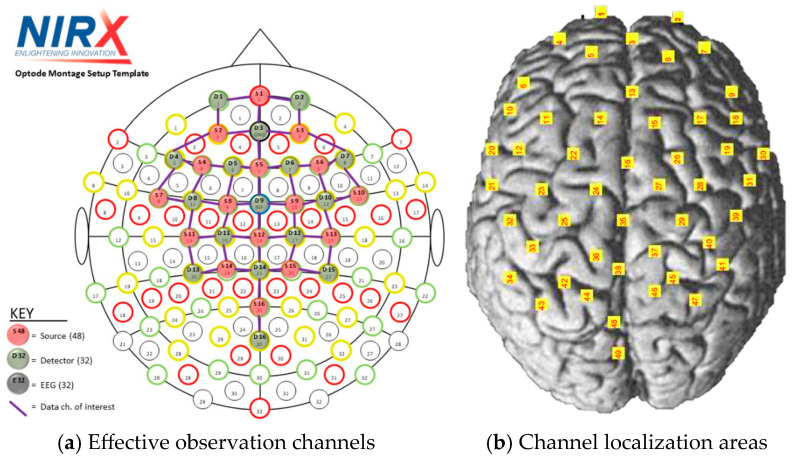
fNIRS channel layout: 16 emitters and 16 detectors were used to form 49 effective observation channels. (© 2026 by the authors).

A multichannel fNIRS system (NIRx, Shanghai, China) operating in continuous-wave mode was used to record brain activity. Optode positions were determined using an NIRS-EEG compatible cap (EASYCAP, Herrsching, Germany) based on the international 10–20 system. The montage comprised 16 sources and 16 detectors, yielding 49 valid measurement channels (Figure 5a). The average source-detector distance was 3.5 cm (range: 2.9–4.1 cm). The layout was designed with NIRsite software (NIRSite 2021.4). Following fNIRS data acquisition, a 3D digitizer was used to identify the Cz, Nz, AL, and AR points and probe locations. Channel positions were co-registered to the Montreal Neurological Institute (MNI) standard space using a probabilistic registration method to estimate their corresponding Brodmann areas. This study focused on the frontal and parietal lobes. The brain regions listed in Figure 5b represent the areas with the highest probability of coverage for each channel. The correspondence between channel layout and Brodmann areas is presented in Table 1.

### 2.5. fNIRS Data Analysis

The fNIRS device used 49 channels with a source–detector distance of 3.5 cm. It measured concentration changes in oxyhemoglobin (HbO) and deoxygenated hemoglobin (HHb) in the frontal and parietal lobes. The system operated at wavelengths of 760 nm and 850 nm, with a differential pathlength factor of 6 and a sampling rate of 10 Hz. HbO was the primary index because HHb correlates highly with signals from other neuroimaging techniques (Huppert et al., 2006), and HbO is more sensitive to task-related neural activity with a higher signal-to-noise ratio.

*Channel quality control.* Pre-experimental channel quality inspection was performed for all 49 recording channels via real-time visual examination using Aurora 1.3 software before formal data acquisition. Pre-recording channel screening was completed in real time via Aurora 1.3 software to exclude defective channels satisfying any of three exclusion criteria: (1) flat signal with an amplitude range lower than 0.1 optical density (OD) units; (2) excessive high-frequency noise with a standard deviation of OD signals exceeding 0.5 within the initial 10 s of baseline recording; (3) persistent motion artifacts characterized by sharp signal spikes greater than 0.5 OD. Data collection commenced only when no fewer than 80% of all channels (i.e., a minimum of 40 channels) exhibited acceptable signal quality. All poor-quality channels were entirely excluded from subsequent preprocessing and statistical analyses, with no signal interpolation applied to preserve data authenticity. For the final sample of 40 participants, the average number of valid channels retained per participant was 44.2 (*SD* = 2.1).

*Data preprocessing.* Raw data were exported to the Homer2 fNIRS processing package (version 2.8; Huppert et al., 2009) and converted to optical density changes. Motion artifacts were corrected using a wavelet filter with an interquartile range (IQR) of 1.5, and a band-pass filter (0.01–0.5 Hz) was applied to remove low-frequency drift and high-frequency noise.

*Physiological noise reduction using PCA.* Principal component analysis (PCA) embedded within the Homer2 hmrDecompPCA function further mitigated widespread systemic physiological artifacts originating from cardiac and respiratory oscillation using the hmrDecompPCA function embedded in Homer2. To capture and eliminate spatially diffuse global artifacts, the continuous fNIRS signal was decomposed within a sliding time-block framework (10 s window length with a 1 s step size). For each time block, the dominant principal components collectively accounting for 97% of the total channel-wise variance were removed. This threshold was selected based on empirical evidence that systemic physiological noise (e.g., cardiac, respiratory, and vasomotor rhythms) typically dominates fNIRS signal variance, loading heavily onto the first few principal components that explain the vast majority (often >95%) of total variance across channels (Santosa et al., 2013; Mehta et al., 2006). The cleaned fNIRS signal was subsequently reconstructed from the remaining low-variance components that preserved task-specific cortical activity. This PCA-based denoising strategy effectively eliminates global, spatially distributed physiological artifacts while retaining localized, task-evoked hemodynamic responses. Following noise reduction, the modified Beer–Lambert law was applied to compute relative changes in oxyhemoglobin (HbO) concentration.

*Event timing and task window.* Each trial started with the presentation of a directional circle stimulus (Figure 3). The stimulus was displayed for up to 4000 ms, and it terminated instantly upon participants’ button-press responses. After each response, a fixation cross appeared for a jittered inter-trial interval (ITI) of 2000–3000 ms (*M* = 2500 ms). The task-related hemodynamic response window was defined as 0–12 s after stimulus onset, covering the entire canonical hemodynamic response function from rise to post-peak return to baseline. The pre-stimulus baseline for each trial was defined as the 2 s interval immediately preceding stimulus onset (−2 to 0 s), which was free of interference from the hemodynamic signals of previous trials. To mitigate potential overlap of hemodynamic responses across consecutive trials, we adopted three strategies: first, we used a jittered ITI (2000–3000 ms) to desynchronize the phase of residual hemodynamic activity between trials; second, we applied band-pass filtering (0.01–0.5 Hz) and PCA-based physiological noise removal to suppress slow drift and lingering hemodynamic tail effects; third, only trials with correct responses were included for averaging, which reduced unstable signal fluctuations caused by error trials. Despite these controls, weak overlap of late-phase hemodynamic responses between adjacent trials could not be completely eliminated, which under the long-ITI design is uncontaminated by preceding responses.

*Trial-level segmentation, averaging, and baseline correction.* We first segmented continuous fNIRS recordings into epochs ranging from −2 s to +14 s relative to stimulus onset. Only trials with correct behavioral responses were included in further analyses. For each participant, measurement channel, and experimental condition (cognitive style × scene layout), we averaged the HbO time series of all valid trials to generate event-related waveforms. Mean HbO concentration within the task window (0–12 s after stimulus onset) was then computed from the averaged waveforms. To ensure robust signal averaging, a minimum of 15 correct trials per condition (out of 24 total trials) was required for participant inclusion. All 40 participants met this criterion. Trial-wise baseline correction was applied to every epoch: the mean HbO value over the 2 s pre-stimulus period (−2 to 0 s) was subtracted from the full post-stimulus time course (0 to 14 s). This approach mitigates low-frequency signal drifts and is widely adopted in event-related fNIRS research. All changes in hemoglobin concentration are presented in micromolar (μM).

*Region-of-interest (ROI) analysis.* Based on the channel-to-brain-region anatomical mapping presented in Table 1 and combined with classical neuroimaging theories as well as the theoretical hypothesis of the current spatial updating study, we further adopted an a priori ROI-level analysis strategy to control for multiple testing biases. Five core frontal–parietal ROIs closely relevant to spatial cognition were predefined in advance: middle frontal gyrus, supplementary motor area, precentral gyrus, postcentral gyrus, and superior parietal lobule. According to the Brodmann area correspondence in Table 1, each ROI included the following channels: (1) middle frontal gyrus: CH06, CH07, CH09, CH11, CH12, CH17, CH18, CH19; (2) supplementary motor area: CH15, CH16; (3) precentral gyrus: CH23, CH25, CH29, CH30, CH31, CH39; (4) postcentral gyrus: CH21, CH32, CH33, CH36, CH37, CH40, CH41, CH45; (5) superior parietal lobule: CH42, CH43, CH44.

Per-channel HbO readings within identical ROIs were averaged to produce a unified ROI-level hemodynamic index for statistical testing. Statistical analyses were primarily performed at the ROI level rather than conducting separate statistical tests for all 49 individual channels. This a priori ROI aggregation was implemented to curtail Type I error inflation driven by excessive single-channel multiple comparisons, effectively avoided Type I error inflation caused by massive channel-wise multiple testing, and improved the statistical robustness, interpretability, and reproducibility of fNIRS neural activation findings. In addition, FDR correction for multiple comparisons (Benjamini–Hochberg procedure, *q* < 0.05) was further applied to the remaining channel-wise results for supplementary verification.

It is important to note that fNIRS spatial localization is approximate. The coverage rates reported in Table 1 and Table 3 indicate the likelihood that a given channel primarily samples from a specific Brodmann area; lower rates suggest greater anatomical ambiguity. Therefore, all brain-region labels should be interpreted as approximate cortical regions rather than precise anatomical points.

Statistical analysis of fNIRS data. A mixed-design ANOVA was conducted using SPSS 20.0, with the significance threshold set at *p* < 0.05 (two-tailed). Effect sizes were estimated with partial eta-squared (*η_p_*^2^). Mauchly’s test was used to assess sphericity, and when the assumption was violated, degrees of freedom were corrected using the Greenhouse–Geisser adjustment. For the channel-wise results that passed the initial ANOVA screening, FDR correction for multiple comparisons (Benjamini–Hochberg procedure, *q* < 0.05) was applied.

## 3. Results

### 3.1. Behavioral Results

Prior to conducting the mixed-design ANOVAs, preliminary assumption checks for parametric testing were performed. For reaction time (RT), residual normality was verified via the Shapiro–Wilk test on the residuals from the full model, yielding *W* = 0.974, *p* = 0.312, indicating no significant deviation from normality. Visual inspection of Q-Q plots and residual histograms supported this conclusion. Homogeneity of variances across the four condition cells (FI-rectangular, FI-oval, FD-rectangular, FD-oval) was tested using Levene’s test, which was non-significant (*F* = 1.324, *p* = 0.276). Sphericity did not apply as the within-subjects factor (scene layout) had only two levels. For accuracy (proportion data), although ANOVA is robust to moderate violations of normality for bounded variables, we conducted a confirmatory generalized linear mixed model (GLMM) with a binomial distribution (logit link) using the lme4 package in R (v1.1 series; Bates et al., 2015). The GLMM confirmed the same pattern of significant effects: a main effect of field cognitive style (χ^2^ = 5.81, *p* = 0.016) and a significant interaction between cognitive style and scene layout (χ^2^ = 4.15, *p* = 0.042), consistent with the ANOVA results reported below. Therefore, the ANOVA findings are reliable and not compromised by assumption violations.

**Figure 6:**
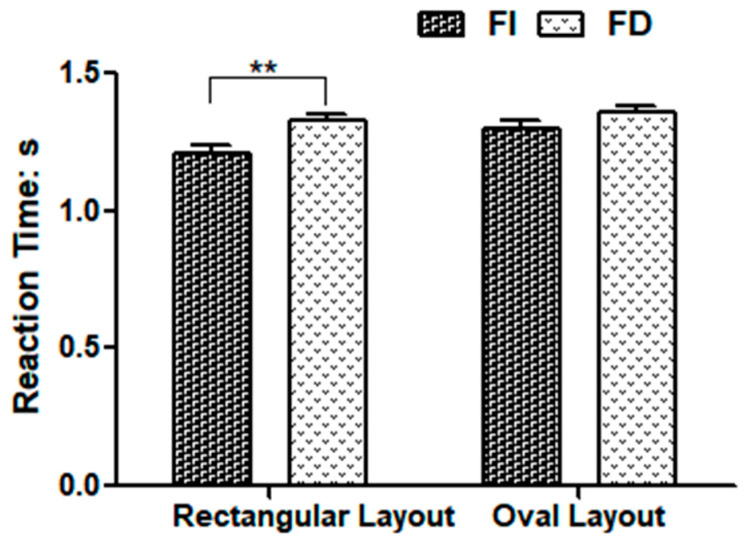
Effect of field cognitive style (FI = field-independent; FD = field-dependent) and scene layout on reaction time, after a natural log transformation to the raw RTs. Error bars indicate standard error. The double asterisk (**) indicates a highly significant difference between FI and FD groups under the rectangular layout condition, *p* < 0.01. (© 2026 by the authors).

**Figure 7:**
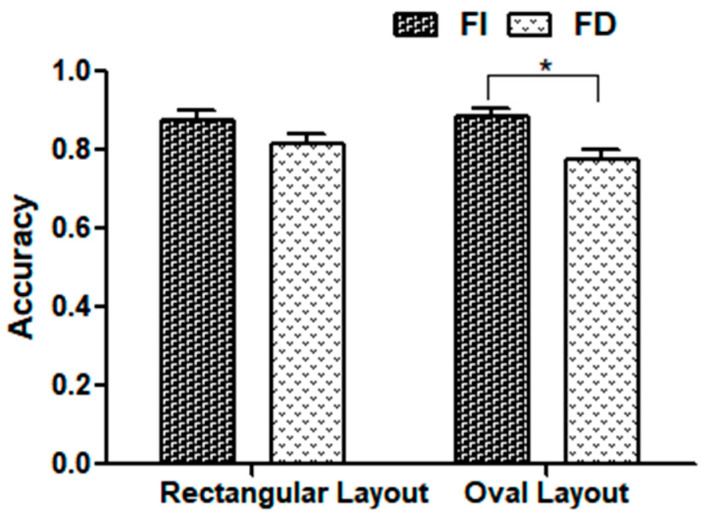
Effect of field cognitive style (FI = field-independent; FD = field-dependent) and scene layout on accuracy. Error bars indicate standard error. The single asterisk (*) indicates a statistically significant difference between FI and FD groups under the oval layout condition, *p* < 0.05. (© 2026 by the authors).

Behavioral results were analyzed by comparing reaction times and accuracy of field-independent and field-dependent individuals performing the spatial updating pointing task in rectangular and oval scene layouts. A repeated-measures ANOVA and simple effect analysis were conducted. Descriptive statistical values for behavioral indicators are summarized in Table 2. It should be noted that the recorded RT encapsulated the full cognitive sequence of reading task requirements, mentally constructing imagined spatial perspectives, completing spatial updating computation, and selecting a response option, rather than only focusing on a limited 12 s trial analysis window. Consistent with conventional reporting norms, standard deviation (*SD*) is listed alongside mean values in Table 2 for raw data description, whereas error bars in Figure 6 and Figure 7 denote standard error (*SE*); this conventional distinction is adopted for visual comparison (*SE*) and raw data description (*SD*).

Owing to inherent positive skewness commonly observed in raw RT distributions, natural logarithmic transformation was applied to all original latency values prior to inferential statistical analysis. Condition means were then recomputed, and a 2 × 2 mixed ANOVA was conducted. For reaction time (RT), the main effect of field cognitive style was significant, *F*(1, 38) = 7.868, *p* = 0.008, *η_p_*^2^ = 0.172, 95% CI [0.025, 0.154], indicating that FI individuals responded significantly faster than FD individuals. The main effect of scene layout was also significant, *F*(1, 38) = 19.103, *p* < 0.001, *η_p_*^2^ = 0.335, 95% CI [0.032, 0.087], indicating that participants responded significantly faster in the rectangular layout than in the oval layout. The interaction between field cognitive style and scene layout was significant (Figure 6), *F*(1, 38) = 6.095, *p* = 0.018, *η_p_*^2^ = 0.138, 95% CI [−0.190, −0.055]. Subsequent simple-effect decomposition revealed that field-independent individuals responded significantly faster than field-dependent individuals in the rectangular layout, *F*(1, 38) = 13.607, *p* = 0.001, *η_p_*^2^ = 0.264, 95% CI [0.055, 0.190].

Accuracy metrics were analyzed via repeated-measures ANOVA, as only aggregated condition-level accuracy ratios rather than trial-wise binary correct/incorrect outcomes were archived. In this study, we only recorded aggregated condition-level accuracy rather than saving binary correct/incorrect responses for every single trial. Therefore, we adopted conventional repeated-measure ANOVA for statistical analysis. For accuracy, the main effect of field cognitive style was significant, *F*(1, 38) = 5.536, *p* = 0.024, *η_p_*^2^ = 0.127, 95% CI [0.012, 0.158], indicating that field-independent individuals achieved significantly higher accuracy than field-dependent individuals. The interaction between field cognitive style and scene layout was significant (Figure 7), *F*(1, 38) = 4.300, *p* = 0.045, *η_p_*^2^ = 0.102, 95% CI [0.033, 0.187]. Further simple effect analysis showed that field-independent individuals achieved significantly higher accuracy than field-dependent individuals in the oval layout, *F*(1, 38) = 8.386, *p* = 0.006, *η_p_*^2^ = 0.181, 95% CI [0.829, 0.938].

### 3.2. fNIRS Results

After implementing a priori frontal–parietal ROI aggregation analysis and FDR multiple comparison correction, the fNIRS results primarily compared HbO concentration changes between field-independent and field-dependent individuals during the spatial updating pointing task in rectangular and oval scene layouts. A repeated-measures ANOVA and simple effect analyses were conducted. Table 3 presents the significant fNIRS results. The detailed results for all channels are presented in Supplementary Table S1.

**Figure 8:**
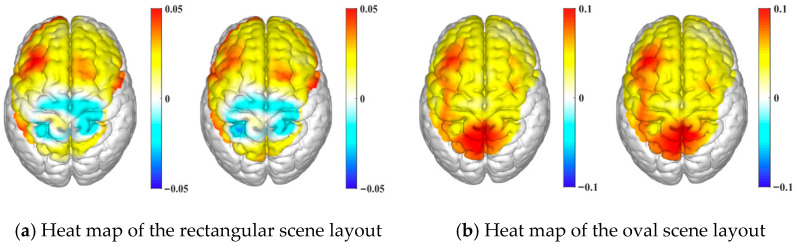
*F*-value heat maps of fNIRS channels for the main effect of scene layout (rectangular vs. oval layout) across all 49 measurement channels. The standardized color bar is presented on the right side of the heat map, with warmer colors (yellow to red) representing higher *F*-values and stronger activation differences, and cooler colors (blue to green) representing lower *F*-values and weaker activation differences. All statistical analyses were performed via mixed-design ANOVA with *df* = 38. The significance level was set at *p* < 0.05 with FDR correction for multiple comparisons. The original brain anatomical backgrounds are replaced with F-value statistical heat maps for intuitive comparison of cortical activation differences. (© 2026 by the authors).

The repeated-measures ANOVA revealed a significant main effect of field cognitive style. Significant activation was observed in channels 23 and 29, corresponding to the precentral gyrus; channel 38, approximately covering the precuneus cortical region with a relatively low coverage rate; channel 44, corresponding to the superior parietal lobule; and channel 46, roughly overlapping with the paracentral lobule at a low coverage level. It is important to note that fNIRS provides approximate cortical localization with limited spatial specificity; the channel-to-Brodmann area correspondences in Table 1 are probabilistic and should not be interpreted as precise anatomical sources. In all these regions, significant HbO elevation within FI relative to FD participants was detected at channels targeting the precentral gyrus, superior parietal lobule, and paracentral lobule. A significant interaction was found between field cognitive style and scene layout. Significant activation was observed in channel 11, corresponding to the middle frontal gyrus. Simple effect analysis further revealed that field-dependent individuals showed significantly higher activation in the rectangular scene compared to the oval scene, *F*(1, 38) = 5.011, *p* = 0.031, *η_p_*^2^ = 0.119, 95% CI [0.004, 0.154]. Outcomes from predefined ROI-level statistical analyses corroborated single-channel corrected findings that the activation differences were mainly concentrated in the predefined fronto-parietal core ROIs, supporting the reliability of the current neural findings. Mean HbO values across all 49 measurement channels under distinct experimental conditions are visualized via cortical *F*-value heatmaps in Figure 8.

## 4. Discussion

The present investigation combined fNIRS with a classic spatial updating paradigm to delineate how field cognitive style and environmental layout jointly modulate spatial updating mechanisms. It focused on behavioral performance differences between FI and FD individuals in rectangular and oval virtual scenes, as well as the underlying fronto-parietal neural activation patterns.

### 4.1. The Modulation of Spatial Updating Performance by Field Cognitive Style and Scene Layout

The behavioral results directly address the first two research questions. Regarding the first, FI individuals exhibited significantly shorter reaction times and higher accuracy than FD individuals across both scene layouts. This finding is consistent with meta-analytic evidence (Boccia et al., 2017) and aligns with prior virtual navigation studies (Nori et al., 2023). Such performance superiority presumably originates from FI individuals’ inherent propensity to adopt internal reference frames when processing environmental information (Witkin, 1977), enabling them to restructure spatial information more efficiently and adapt to changing perspectives.

The present behavioral results confirmed consistent cognitive style advantages across spatial updating tasks: FI individuals exhibited faster reaction times and higher accuracy overall compared with FD individuals, which supports previous meta-analytic findings on field independence and spatial navigation performance. This dissociation provides novel insights into how the benefits of cognitive style manifest differently depending on the environmental demand. The manipulation check results further validated our environmental geometry manipulation: participants clearly perceived prominent global directional axes in the rectangular layout, whereas the oval layout was rated as lacking stable global reference frames, even with identical local landmark objects in both environments.

The reaction time advantage of FI individuals in the rectangular layout can be interpreted through reference frame alignment. Rectangular rooms offer salient orthogonal axes (He et al., 2018). FI individuals excel at extracting these geometric cues, which may facilitate rapidly aligning internal spatial representations with environmental axes, potentially accelerating directional judgments. By contrast, FD individuals depend more heavily on external cues and require more time to establish correspondence between the egocentric perspective and the allocentric frame (Zhu et al., 2022). This pattern is consistent with earlier findings (Tascón et al., 2017). It further clarifies that the FI advantage is most pronounced when the environment provides a clear geometric scaffold.

The accuracy advantage of FI individuals in the oval layout reveals a different facet of cognitive style effects. Oval environments, characterized by a continuously curved perimeter without planar wall segments or sharp corners, lack well-defined intrinsic reference axes (Kelly et al., 2008). In such geometrically ambiguous environments, it is plausible that FI individuals can compensate for the absence of salient allocentric cues by flexibly relying on their internal reference frames, as suggested by their preserved accuracy. FD individuals, however, are disproportionately impaired when the external environment fails to offer unambiguous directional anchors. Their heavy reliance on external structure means that when this structure is impoverished, they lose the primary basis for organizing spatial information. This results in significantly lower accuracy. This interpretation is consistent with Nori et al. (2023), who found that FI individuals show a performance advantage when the imagined perspective is far from the body axis—a condition that is inferred to require flexible internal manipulation of spatial representations.

The main effect of scene layout on accuracy was not significant. This null effect likely reflects that the accuracy deficit of FD individuals in the oval layout was partially offset by the stable, high accuracy of FI individuals across both layouts, underscoring the importance of considering individual differences when evaluating environmental geometry. Reaction time was consistently shorter in the rectangular layout, aligning with the facilitative role of geometric cues in spatial orientation (Wolbers et al., 2011). This suggests that environmental geometry may exert a stronger influence on processing speed than on response precision when individual cognitive styles are not considered.

### 4.2. Neural Correlates of Spatial Updating as a Function of Cognitive Style and Scene Layout

fNIRS results revealed that the main effect of field cognitive style was significantly higher activation in FI individuals across multiple brain regions. These included the precentral gyrus (CH23, CH29), precuneus (CH38), superior parietal lobule (CH44), and paracentral lobule (CH46). Given the probabilistic nature of fNIRS spatial registration (see Table 1 coverage rates), these regional labels should be interpreted as approximate cortical areas rather than precise anatomical localizations. It should be noted that the activation of CH38 and CH46 exhibited relatively low coverage rates to the precuneus and paracentral lobule respectively; therefore, their neural interpretations are regarded as approximate cortical activation patterns rather than precise anatomical localizations. The study found that the posterior parietal cortex maintains egocentric spatial processing (Chokron, 2003), while the precuneus supports the encoding of the environment relative to the self (Byrne et al., 2007; Dordevic et al., 2022). While this broad pattern is consistent with the recruitment of egocentric spatial processing networks, it is important to consider complementary interpretations. Nevertheless, these cortical territories are also implicated in spatial attention allocation and high-precision sensorimotor transformation. Therefore, enhanced HbO reactivity among FI individuals may reflect amplified allocation of cognitive resources, which ultimately yields their superior behavioral performance. Conversely, the relatively lower activation in FD individuals could stem from a partial mismatch between their preferred cue-dependent strategy and the task’s requirement to operate on internal representations—a mismatch that may manifest as reduced neural efficiency rather than simply weaker engagement. This finding aligns with Blouin et al. (2022), who identified the right superior posterior parietal cortex and precuneus as key regions involved in spatial updating.

The significant interaction effects observed in the middle frontal gyrus (CH11), provide direct neural evidence for the differential strategies adopted by the two cognitive style groups under varying environmental conditions. In the middle frontal gyrus, FD individuals exhibited significantly higher activation in the rectangular layout compared to the oval layout. The middle frontal gyrus has been implicated in integrating spatial information from multiple sensory channels to form unified spatial representations (Blouin et al., 2022; Chen et al., 2023). Heightened middle frontal hemodynamic reactivity among FD individuals within rectangular settings corresponds to their habitual reliance on externally anchored spatial processing, although causal claims cannot be made from fNIRS data. The salient orthogonal axes provided by rectangular geometry enable FD individuals to actively integrate visual boundary cues with landmark configurations. This facilitates their spatial updating. This interpretation is consistent with the behavioral finding, suggesting that they can capitalize on environmental structure when it is available. In the oval layout, the paucity of external reference cues limits the extent to which FD individuals can engage in this integration hub. This results in reduced middle frontal gyrus activation, a neural pattern that mirrors the behavioral decline observed in FD accuracy under the oval condition. Collectively, this interactive activation pattern suggests that FI individuals may engage in more flexible fronto-parietal dynamics, whereas FD individuals’ neural activity appears more tightly coupled to the availability of environmental structure.

Overall, the present neuroimaging outcomes corroborate the theoretical framework proposed by Zhao and Warren (2024), who emphasized that spatial updating depends on the flexible integration of multiple reference frames rather than reliance on a single frame. The differential activation patterns observed here are interpreted to indicate that the manner in which these frames are weighed is jointly determined by the individual’s cognitive style and the structural properties of the environment. FI individuals possess a functionally adaptable fronto-parietal control network (FPN) supporting cognitive flexibility, which we infer enables the efficient reconfiguration between externally oriented attention and internally driven processing (Cole et al., 2013). In the context of our scene layout task, the FPN is critically involved in top-down attentional control, including orienting attention to task-relevant spatial cues while filtering out misleading background structures (Corbetta & Shulman, 2002), and resolving conflicts between internal cognitive schemas and external perceptual input (Vincent et al., 2008). This interpretation aligns with previous evidence showing differential FPN engagement between FI and FD individuals during tasks requiring the suppression of salient but irrelevant contexts (Heinzel et al., 2013; Derbie et al., 2021). In contrast, FD individuals’ neural activity is more tightly coupled to the availability of external environmental structure. This stronger coupling likely reflects a reduced capacity for top-down modulation in the absence of clear environmental cues, consistent with the context-updating function attributed to the FPN (Boccia et al., 2017; Nori et al., 2023).

### 4.3. Theoretical and Practical Implications

The present findings refine theoretical frameworks by specifying one pathway through which cognitive style influences spatial updating: our data can be interpreted to suggest that FI individuals may flexibly recalibrate reliance on internal versus external reference frames depending on environmental demands, whereas FD individuals perform adequately only when a clear allocentric scaffold is available. This characterization extends prior work (Nori et al., 2023) by identifying neural correlates of these divergent processing styles during dynamic spatial updating. From an applied perspective, these results only provide tentative and preliminary references for adaptive human–machine interfaces and architectural spatial layout planning. Given that the current experiment adopted a pure screen-based paradigm without real proprioceptive and vestibular movement cues, the practical application value of the findings is limited and needs to be verified in real scenarios. Establishing clearly defined environmental reference systems through orthogonal architectural axes, salient boundary cues, or stabilized directional indicators may help reduce disorientation and cognitive load at the source. It is speculated that such environmental design strategies may yield more benefits to individuals who rely heavily on external spatial cues, without substantially impairing the orientation efficiency of those who prefer internal reference processing. Through intelligent environments that dynamically adjust cue salience, spatial updating support could eventually become more personalized and adaptive. Nonetheless, direct practical translation awaits further empirical validation via immersive full-body virtual reality or in situ real-world navigational assessments.

### 4.4. Limitations and Future Directions

This study has several limitations. First, the fNIRS probe arrangement exclusively sampled frontal and parietal cortices, failing to cover temporal and occipital regions functionally implicated in scene perceptual processing (Kang, 2023; Li et al., 2023). Future research could adopt whole-head fNIRS to reveal more complete neural mechanisms. Second, the sample consisted solely of university students selected based on extreme EFT scores, while this extreme-group strategy effectively amplified cognitive style differences and is consistent with prior practice (Boccia et al., 2017; Nori et al., 2023). While the study recruited only typical FI and FD participants without including intermediate cognitive style individuals, which restricts the extrapolation of our results to the entire population along the FDI continuum, future research should adopt a continuous or stratified sampling strategy to cover intermediate FDI individuals and verify the replicability and generalizability of the findings. Although power analysis validated our minimum sample and we obtained significant interactions, the 40-participant sample is modest owing to extreme-group sampling, limiting result generalizability. To address the limitation of small sample size, future research should further expand the sample size, recruit non-student groups and participants with intermediate FDI scores to improve the external validity of the conclusions. Third, the task adopted screen-based virtual presentation without real proprioceptive and vestibular movement cues (Allison & Head, 2017), which may amplify cognitive style differences in visual–spatial working memory. Future studies using immersive virtual reality or real-world navigation paradigms could examine whether the provision of body-based cues attenuates or modifies the interaction between cognitive style and environmental geometry. Fourth, although we recorded video repetitions and confirmed no group difference in learning exposure in the current study, we did not further examine whether individual replay frequency continuously modulated behavioral accuracy or prefrontal-parietal activation. Future research should treat video replay times as a covariate in statistical modeling to more strictly control individual differences in learning efficiency. Fifth, a significant analytical constraint of the present study is that our fNIRS montage did not include short-separation (SS) channels (i.e., source-detector distance < 1 cm), which are optimal for regressing out superficial physiological noise such as scalp blood flow and cardiac pulsations (Brigadoi & Cooper, 2015). Consequently, despite our application of PCA-based denoising to suppress global physiological artifacts, we cannot rule out the possibility that some portion of the HbO signals reflects superficial hemodynamic changes rather than genuine cortical task-evoked responses. This limitation is inherent to our montage design and may affect the specificity of the reported activation patterns. For future studies, multi-distance optode placement and short-separation channels should be integrated to better isolate genuine cortical task-evoked signals. In addition, given the 2000–3000 ms jittered ITI and the 0–12 s hemodynamic analysis window adopted in the event-related fNIRS design, late components of hemodynamic responses may still produce mild cross-trial signal overlap, even after signal filtering and noise reduction. This is an inherent limitation of the current paradigm. In future studies, extending the ITI to over 4000 ms or adopting block-design fNIRS paradigms could further reduce hemodynamic overlap between trials. Sixth, another inherent analytical constraint is that only aggregated condition-level accuracy was archived rather than trial-level binary correct/incorrect responses. This precludes more rigorous trial-based logistic mixed-effects modeling, which could have accounted for trial-by-trial variability and provided more precise estimates of condition effects on accuracy. As a result, our accuracy analyses are limited to condition-level aggregates and may be less sensitive to subtle performance fluctuations across trials. Future studies should preserve individual trial correct/incorrect responses to enable logistic mixed-effects modeling for bounded accuracy data. Finally, the original purely channel-wise statistical analysis across 49 channels lacked strict multiple comparison control, which might lead to unstable marginal significant results near the *p* = 0.05 threshold. In the revised version, we adopted a priori ROI aggregation analysis combined with FDR correction to control statistical bias effectively. Future fNIRS studies on spatial cognition are recommended to predefine theoretical ROIs based on anatomical channel mapping, prioritize ROI-level statistical inference, and apply multiple comparison correction such as FDR to enhance result credibility.

## 5. Conclusions

This study systematically investigated the behavioral and neural mechanisms through which field cognitive style and environmental scene geometry jointly modulate spatial updating. Behavioral results revealed that FI participants exhibited significantly shorter response latencies (*M* = 18.75 s) and higher overall task accuracy (*M* = 88.50%) compared with FD participants (*M* = 22.75 s, 79.50%). Environments equipped with explicit allocentric geometric reference frames (rectangular layout) induced faster responses across all participants (*M* = 19.75 s) compared with oval settings devoid of stable global directional axes (*M* = 21.75 s), whereas overall accuracy was comparable between the two scene types (rectangular: 84.50%; oval: 83.50%). A significant interaction emerged between cognitive style and scene layout: within geometrically ambiguous oval environments with scarce extrinsic directional cues, FD participants showed marked declines in accuracy (78.00%) and prolonged reaction times (23.00 s). In contrast, FI individuals maintained stable spatial updating performance irrespective of environmental geometric integrity (accuracy: 89%; response latency: 20.5 s). These divergent behavioral patterns are interpreted to demonstrate that FI individuals flexibly recruit internal egocentric reference frames when environmental geometric anchors are unavailable, whereas FD performance is markedly compromised in the absence of salient environmental structural cues. Corresponding fNIRS hemodynamic results further revealed interactive cortical activation disparities localized to the middle frontal gyrus: FD participants exhibited significantly elevated oxyhemoglobin (HbO) activation within rectangular relative to oval configurations, reflecting intensified neural engagement during processing of available external environmental geometry. These neurophysiological discrepancies corroborate the critical functional contributions of both egocentric and allocentric reference systems to spatial updating, with their relative weighting dynamically shaped by individual field cognitive style. Collectively, the present findings can serve as limited empirical references for the rational design of environmental reference systems to mitigate spatial disorientation and excessive cognitive load, although the screen-based task cannot fully simulate real bodily movement and sensory cues in natural navigation. Targeted structural optimization of built surroundings may help reduce orientation difficulties among FD individuals without compromising spatial orientation efficiency for FI individuals. Following further validation through immersive virtual reality paradigms and real-world navigation experiments, these results could potentially serve as a limited reference for the development of personalized intelligent navigation prompts and human-centered adaptive spatial interface systems, which may help improve spatial orientation performance in related scenarios.

## Figures and Tables

**Table 1:** Correspondence between channel layout and Brodmann areas.

	Channel Layout
Gyri Frontalis Superior	CH01, CH02, CH04, CH05, CH08, CH14, CH22, CH24, CH26, CH27, CH28
Medial Part of the Superior Frontal Gyrus	CH03, CH13
Middle Frontal Gyrus	CH06, CH07, CH09, CH11, CH12, CH17, CH18, CH19
Triangular Part of the Inferior Frontal Gyrus	CH10
Supplementary Motor Area	CH15, CH16
Opercular Part of the Inferior Frontal Gyrus	CH20
Postcentral Gyrus	CH21, CH32, CH33, CH36, CH37, CH40, CH41, CH45
Precentral Gyrus	CH23, CH25, CH29, CH30 CH31, CH39
Inferior Parietal Lobe	CH34
Paracentral Lobule	CH35, CH46, CH47
Precuneus	CH38, CH48, CH49
Superior Parietal Lobe	CH42, CH43, CH44

The layout of these channels in the frontal and parietal brain regions was measured, and the positions of Cz, Nz, AL, AR points and probes were determined using a 3D positioning instrument. The position of the fNIRS channel is matched with the MNI spatial coordinates through the probabilistic registration method to obtain the information between the FNIRS channel and the Brodmann partition correspondence.

**Table 2:** Descriptive statistics of behavioral data. Mean (*M*) and standard deviation (*SD*) of reaction time and accuracy in the scene layout and field cognitive style (N = 40).

Performance	Scene Layout	Field-Independent		Field-Dependent	
		*M*	*SD*	*M*	*SD*
Reaction Time	Rectangular	16.773	4.576	21.844	4.102
	oval	20.769	5.370	23.333	5.112
Accuracy	Rectangular	0.874	0.118	0.814	0.123
	oval	0.884	0.107	0.774	0.132

*M* (mean) and *SD* (standard deviation) are retained to three decimal places.

**Table 3:** Significant fNIRS results in 49 channels under various conditions.

Condition	Channel	Spatial Coordinates	Brain Region	Coverage Rate	*F*	*p*	*η_p_* ^2^	*95% CI*
Field Cognitive Style	CH23	(−38, −6, 65)	Precentral Gyrus	86.84%	5.504	0.024	0.129	−0.114, −0.006
	CH29	(23, −20, 76)	Precentral Gyrus	91.00%	6.259	0.017	0.145	−0.117, −0.016
	CH38	(−3, −43, 76)	Precuneus	46.23%	5.362	0.026	0.127	−0.135, −0.007
	CH44	(−18, −55, 74)	Superior Parietal Lobule	72.54%	4.801	0.035	0.115	−0.217, −0.010
	CH46	(13, −52, 77)	Paracentral Lobule	42.22%	5.070	0.030	0.121	−0.198, −0.018
Field Cognitive Style × Scene Layout	CH11	(−35, 25, 55)	Middle Frontal Gyrus	100.00%	6.437	0.016	0.148	−0.122, 0.043

The HbO concentration combined with the registration method corresponds to the detection data to the MNI spatial coordinates. By comprehensively measuring the position of the probe and mapping it to the brain model, the range of the detection area is determined, and the coverage rate is obtained. All *p*-values were corrected by FDR multiple comparison correction; only channels with significant activation after FDR correction are retained. Marginal significant channels (0.039 < *p* < 0.05) that failed to pass FDR correction were excluded. Notably, some channels in Table 3 showed relatively low probabilistic regional coverage (e.g., Channel 38, Precuneus, 46.23%; Channel 46, Paracentral Lobule, 42.22%). Low anatomical coverage values (below 50%, e.g., CH38, CH46) denote imprecise regional mapping, and such results should only be interpreted as approximate cortical activation.

## Data Availability

The data presented in this study are available on request from the corresponding author. The data are not publicly available due to privacy concerns.

## Supplementary Materials

The following supporting information can be downloaded at: https://www.mdpi.com/article/10.3390/bs16071125/s1, Table S1: Comparison of Field-Independent (FI) and Field-Dependent (FD) Conditions Across Brain Regions and Scene Layouts.

## Abbreviations

The following abbreviations are used in this manuscript:

fNIRS	functional near-infrared spectroscopy
JRD	Judgment of relative direction
EFT	Embedded Figures Test
ANOVA	Analysis of Variance
PCA	Principal component analysis
IQR	Interquartile range
FDI	Field dependence–independence
FI	Field-independent
FD	Field-dependent
RT	Reaction time
HbO	Oxyhemoglobin
HHb	Deoxygenated hemoglobin
SMA	Supplementary motor area
MFG	Middle frontal gyrus
MNI	Montreal Neurological Institute
