# Supplementary material for: The Spatial Updating Mechanism of Different Field Cognitive Styles in Various Scene Layouts: Evidence from Behavior and fNIRS
Source: Behav Sci (Basel). 2026 Jul 6;16(7):1125. doi: 10.3390/bs16071125 (PMC13405959; doi:10.3390/bs16071125)
Supplement: Supplementary file 1 [file behavsci-16-01125-s001.zip › behavsci-4327592-supplementary.pdf]

Supplementary Table S1:

Comparison of Field-Independent (FI) and Field-Dependent (FD) Conditions Across Brain Regions and Scene Layouts

Brain Region	Channel	Scene Layout	Field-Independent		Field-Dependent		Effect Direction	FDR-corrected *q*
			M	SD	M	SD		
Gyri frontalis superior	CH01	Rectangular	0.043	0.111	0.054	0.162	FI<FD	0.994
		Oval	0.043	0.105	0.044	0.148	FI>FD	0.917
	CH02	Rectangular	0.056	0.207	-0.004	0.229	FI>FD	0.369
		Oval	0.055	0.174	0.012	0.225	FI>FD	0.418
	CH04	Rectangular	0.028	0.078	0.032	0.155	FI<FD	0.945
		Oval	0.026	0.068	0.022	0.134	FI>FD	0.970
	CH05	Rectangular	0.047	0.102	0.012	0.084	FI>FD	0.255
		Oval	0.042	0.109	0.021	0.069	FI>FD	0.351
	CH08	Rectangular	0.018	0.100	0.021	0.118	FI>FD	0.970
		Oval	0.019	0.102	0.018	0.105	FI>FD	0.931
	CH14	Rectangular	0.055	0.090	0.021	0.081	FI>FD	0.180
		Oval	0.057	0.080	0.018	0.074	FI>FD	0.132
	CH22	Rectangular	0.043	0.097	0.015	0.070	FI>FD	0.252
		Oval	0.051	0.103	0.011	0.067	FI>FD	0.174
	CH24	Rectangular	0.045	0.085	-0.002	0.096	FI>FD	0.074
		Oval	0.049	0.079	-0.004	0.092	FI>FD	0.055
	CH26	Rectangular	0.042	0.068	0.037	0.114	FI>FD	0.972
		Oval	0.052	0.104	0.042	0.127	FI>FD	0.903
	CH27	Rectangular	0.047	0.084	0.007	0.099	FI>FD	0.145
		Oval	0.055	0.090	0.010	0.094	FI>FD	0.122
	CH28	Rectangular	0.075	0.114	0.011	0.113	FI>FD	0.133
		Oval	0.070	0.119	0.016	0.110	FI>FD	0.173
Medial Part of the Superior Frontal Gyrus	CH03	Rectangular	0.036	0.071	0.014	0.137	FI>FD	0.515
		Oval	0.039	0.067	0.020	0.128	FI>FD	0.535
	CH13	Rectangular	0.045	0.062	0.024	0.060	FI>FD	0.190

		Oval	0.051	0.054	0.023	0.049	FI>FD	0.109
	CH06	Rectangular	0.047	0.083	0.030	0.101	FI>FD	0.550
		Oval	0.046	0.080	0.031	0.080	FI>FD	0.532
	CH07	Rectangular	0.015	0.089	0.011	0.175	FI>FD	0.953
		Oval	0.012	0.091	0.031	0.190	FI<FD	0.851
	CH09	Rectangular	0.023	0.102	0.024	0.098	FI>FD	0.975
		Oval	0.021	0.101	0.020	0.096	FI>FD	0.950
	CH11	Rectangular	0.093	0.128	0.054	0.126	FI > FD	0.286
		Oval	0.102	0.145	0.040	0.112	FI>FD	0.183
Middle Frontal Gyrus	CH12	Rectangular	0.069	0.078	0.039	0.081	FI>FD	0.257
		Oval	0.078	0.086	0.036	0.073	FI>FD	0.172
	CH17	Rectangular	0.063	0.106	0.019	0.094	FI>FD	0.188
		Oval	0.053	0.127	0.013	0.076	FI>FD	0.208
	CH18	Rectangular	0.021	0.089	0.015	0.090	FI>FD	0.784
		Oval	0.027	0.081	0.011	0.085	FI>FD	0.661
	CH19	Rectangular	0.051	0.090	0.018	0.091	FI>FD	0.285
		Oval	0.047	0.093	0.020	0.076	FI>FD	0.333
Triangular Part of the Inferior Frontal Gyrus	CH10	Rectangular	0.036	0.085	0.020	0.065	FI>FD	0.493
		Oval	0.041	0.085	0.021	0.059	FI>FD	0.429
Supplementary Motor Area	CH15	Rectangular	0.038	0.098	0.037	0.095	FI>FD	0.828
		Oval	0.049	0.109	0.029	0.085	FI>FD	0.604
	CH16	Rectangular	0.024	0.046	0.027	0.064	FI>FD	0.896
		Oval	0.033	0.059	0.025	0.065	FI>FD	0.693
Opercular Part of the Inferior Frontal Gyrus	CH20	Rectangular	0.044	0.112	0.032	0.083	FI>FD	0.798
		Oval	0.042	0.112	0.043	0.076	FI>FD	0.965
Postcentral Gyrus	CH21	Rectangular	0.049	0.067	0.007	0.080	FI>FD	0.094
		Oval	0.048	0.061	0.020	0.073	FI>FD	0.139
	CH32	Rectangular	0.020	0.125	0.003	0.055	FI>FD	0.610
		Oval	0.028	0.128	0.016	0.071	FI>FD	0.678

	CH33	Rectangular	0.073	0.138	0.000	0.101	FI>FD	0.070
		Oval	0.081	0.141	-0.007	0.104	FI>FD	0.049
	CH36	Rectangular	0.053	0.072	0.017	0.086	FI>FD	0.171
		Oval	0.054	0.063	0.009	0.092	FI>FD	0.125
	CH37	Rectangular	0.047	0.107	-0.011	0.118	FI>FD	0.124
		Oval	0.052	0.111	-0.003	0.121	FI>FD	0.139
	CH40	Rectangular	0.050	0.089	-0.015	0.110	FI>FD	0.039
		Oval	0.055	0.101	-0.018	0.110	FI>FD	0.034
	CH41	Rectangular	0.023	0.096	0.036	0.063	FI<FD	0.748
		Oval	0.035	0.094	0.026	0.070	FI>FD	0.929
	CH45	Rectangular	0.060	0.098	-0.033	0.183	FI>FD	0.060
		Oval	0.065	0.093	-0.032	0.182	FI>FD	0.053
	CH23	Rectangular	0.074	0.072	0.013	0.092	FI>FD	0.024
		Oval	0.073	0.080	0.010	0.085	FI>FD	0.021
	CH25	Rectangular	0.028	0.078	-0.014	0.079	FI>FD	0.067
		Oval	0.032	0.084	-0.014	0.083	FI>FD	0.063
	CH29	Rectangular	0.046	0.077	-0.020	0.079	FI>FD	0.022
		Oval	0.046	0.081	-0.015	0.087	FI>FD	0.035
Precentral Gyrus	CH30	Rectangular	0.047	0.138	0.043	0.117	FI=FD	0.996
		Oval	0.037	0.106	0.040	0.102	FI<FD	0.915
	CH31	Rectangular	0.029	0.087	0.050	0.103	FI<FD	0.615
		Oval	0.033	0.083	0.049	0.092	FI<FD	0.650
	CH39	Rectangular	0.037	0.126	0.020	0.091	FI>FD	0.515
		Oval	0.041	0.127	0.020	0.089	FI>FD	0.478
Inferior Parietal Lobe	CH34	Rectangular	0.066	0.142	0.048	0.085	FI>FD	0.666
		Oval	0.066	0.134	0.041	0.080	FI>FD	0.595
	CH35	Rectangular	0.019	0.080	-0.018	0.086	FI>FD	0.133
Paracentral Lobule		Oval	0.023	0.077	-0.015	0.082	FI>FD	0.119
	CH46	Rectangular	0.088	0.157	-0.004	0.127	FI>FD	0.038

		Oval	0.102	0.153	-0.006	0.124	FI>FD	0.023
	CH47	Rectangular	0.054	0.085	0.028	0.077	FI>FD	0.348
		Oval	0.056	0.076	0.018	0.078	FI>FD	0.235
	CH38	Rectangular	0.083	0.092	0.011	0.104	FI>FD	0.046
		Oval	0.080	0.089	0.006	0.109	FI>FD	0.047
Precuneus	CH48	Rectangular	0.102	0.268	0.025	0.137	FI>FD	0.284
		Oval	0.100	0.282	0.024	0.111	FI>FD	0.287
	CH49	Rectangular	0.075	0.206	0.002	0.125	FI>FD	0.224
		Oval	0.074	0.199	0.031	0.113	FI>FD	0.359
	CH42	Rectangular	0.034	0.140	-0.030	0.123	FI>FD	0.142
		Oval	0.040	0.140	-0.039	0.122	FI>FD	0.100
Superior Parietal Lobe	CH43	Rectangular	0.062	0.122	0.013	0.122	FI>FD	0.253
		Oval	0.070	0.123	0.009	0.119	FI>FD	0.201
	CH44	Rectangular	0.100	0.190	-0.014	0.125	FI>FD	0.045
		Oval	0.090	0.172	-0.007	0.109	FI>FD	0.054
Middle Frontal Gyrus	CH11	FI-Rectangular	0.067	0.029	0.016	0.034	FD-Rectangular > FD-Oval	0.148
		FI-Oval	0.071	0.032				
		FD-Rectangular	0.098	0.036				
		FD-Oval	0.063	0.030				
